# Temporary Visual Deprivation Causes Decorrelation of Spatiotemporal Population Responses in Adult Mouse Auditory Cortex

**DOI:** 10.1523/ENEURO.0269-19.2019

**Published:** 2019-12-09

**Authors:** Krystyna Solarana, Ji Liu, Zac Bowen, Hey-Kyoung Lee, Patrick O. Kanold

**Affiliations:** 1 Department of Biology, University of Maryland, College Park, MD 20742; 2 Department of Neuroscience, Mind/Brain Institute, Johns Hopkins University, Baltimore, MD 21218

**Keywords:** auditory cortex, cross-modal, dark exposure, plasticity, visual deprivation

## Abstract

Although within-modality sensory plasticity is limited to early developmental periods, cross-modal plasticity can occur even in adults. *In vivo* electrophysiological studies have shown that transient visual deprivation (dark exposure, DE) in adult mice improves the frequency selectivity and discrimination of neurons in thalamorecipient layer 4 (L4) of primary auditory cortex (A1). Since sound information is processed hierarchically in A1 by populations of neurons, we investigated whether DE alters network activity in A1 L4 and layer 2/3 (L2/3). We examined neuronal populations in both L4 and L2/3 using *in vivo* two-photon calcium (Ca^2+^) imaging of transgenic mice expressing GCaMP6s. We find that one week of DE in adult mice increased the sound evoked responses and frequency selectivity of both L4 and L2/3 neurons. Moreover, after DE the frequency representation changed with L4 and L2/3 showing a reduced representation of cells with best frequencies (BFs) between 8 and 16 kHz and an increased representation of cells with BFs above 32 kHz. Cells in L4 and L2/3 showed decreased pairwise signal correlations (SCs) consistent with sharper tuning curves. The decreases in SCs were larger in L4 than in L2/3. The decreased pairwise correlations indicate a sparsification of A1 responses to tonal stimuli. Thus, cross-modal experience in adults can both alter the sound-evoked responses of A1 neurons and change activity correlations within A1 potentially enhancing the encoding of auditory stimuli.

## Abstract (precis)

Although within-modality sensory plasticity is limited to early developmental periods, cross-modal plasticity can occur even in adults. *In vivo* electrophysiological studies have shown that transient visual deprivation (dark exposure, DE) in adult mice improves the frequency selectivity and discrimination of neurons in thalamorecipient layer 4 (L4) of primary auditory cortex (A1).

## Significance Statement

Our results show that the brief period of visual deprivation in mice can alter the tone evoked responses of neurons as well as the frequency representation in multiple layers of the primary auditory cortex (A1). Thus, the tuning of auditory cortex neurons can be altered even after the critical period. Moreover, our results show that pairwise correlations are decreased indicating a sparsification of the evoked responses in the auditory cortex. These results add to the mounting evidence that cross-modal sensory experience has the power to alter network circuitry and population dynamics even into adulthood.

## Introduction

A hallmark of sensory cortices is their ability to rewire in response to environmental input especially during critical periods in development (Wiesel and Hubel, 1963; Hubel and Wiesel, 1970; de Villers-Sidani et al., 2007; Sanes and Bao, 2009; Barkat et al., 2011). Loss of a sensory modality can engage plasticity in the remaining senses in a compensatory manner. Humans experiencing vision loss from birth exhibit cross-modal perceptual enhancement of hearing, including improved sound localization abilities (Lessard et al., 1998; Voss et al., 2004), frequency discrimination performance (Gougoux et al., 2004), and auditory spatial tuning (Röder et al., 1999). Cats binocularly deprived from birth and juvenile ferrets with binocular eye suture both show a significant compensatory enhancement in auditory spatial acuity, particularly in peripheral sound localization (Rauschecker and Kniepert, 1994; King and Parsons, 1999). Remarkably, the capacity for cross-modal induced plasticity is not fully extinguished in adulthood, but the circuit basis for these perceptual enhancements is unclear. Following prolonged visual deprivation, adult ferrets also show improved peripheral auditory spatial acuity (King and Parsons, 1999). Compensatory plasticity in the auditory domain is not only limited to early onset or prolonged vision loss, but can be observed when vision loss occurs later in life and over a shorter period. In humans, late-onset blindness can enhance auditory localization (Voss et al., 2004), and even brief periods of visual deprivation can transiently improve auditory perception by enhancing sound source segregation (Pagé et al., 2016).

On a cellular level, brief periods of visual deprivation in rodents (dark exposure, DE) after the critical period in A1 increase frequency selectivity, lower thresholds, and increase neuronal firing rates of single neurons in L4 of A1 (Petrus et al., 2014). These changes on the single cell level likely result from altered neuronal circuits. Indeed, DE induces potentiation of thalamic input to L4, strengthening of ascending intracortical connections from L4 to L2/3 neurons (Goel et al., 2006; Petrus et al., 2014, 2015), refinement of both excitatory and inhibitory intralaminar connections within L2/3 as well as interlaminar ascending connections from L4 to L2/3 (Meng et al., 2015) and feedback connections from L2/3 to L4 (Meng et al., 2017a). These circuit changes are consistent with the observed changes in L4 responses *in vivo*. However, sensory stimuli are not entirely encoded by single neurons but by populations of neurons and the network activity patterns as well as activity correlations between neurons can contribute to information encoding (Averbeck et al., 2006). Thus, changes in synaptic function must be coordinated across neurons to improve network function.

Since the representation of sound frequency preference differs between L4 and L2/3 of A1 with L2/3 showing more heterogeneous organization than L4 (Bandyopadhyay et al., 2010; Rothschild et al., 2010; Winkowski and Kanold, 2013; Kanold et al., 2014; Maor et al., 2016), we examined whether DE in adulthood can restructure the mesoscale organization and connectivity of A1 using *in vivo* two-photon calcium (Ca^2+^) imaging. We measured the sound evoked activity from L2/3 and L4 A1 neurons in adult mice following one week of DE which was initiated after the critical period for spectral tuning (>P21). We observed that single cells in both L4 and L2/3 show increased frequency selectivity. However, we found that after DE fewer neurons preferred tones in the mid-frequency region. Moreover, we find that after DE activity correlations between local neurons in both L4 and L2/3 were reduced indicating a decorrelation of the population activity in A1. These experiments reveal that besides altering the tuning of single neurons, DE can alter network activity and population dynamics in adulthood, long after the canonical critical period for auditory and visual plasticity has ended (Goel et al., 2006; Barkat et al., 2011; Petrus et al., 2014, 2015; Meng et al., 2015). Thus, cross-modal plasticity might be more powerful than within-modality plasticity in rewiring cortical circuits. Moreover, given that visual deprivation is easily established, cross-modal plasticity could potentially be used for targeted modifications of A1.

## Materials and Methods

To study the cross-modal plasticity of A1 we used *in vivo* two-photon Ca^2+^ imaging in 15 in-house bred male and female *Thy1*-GCaMP6s (GP4.3) transgenic mice (JAX strain 024275; Dana et al., 2014; normal-reared, NR, *n* = 9, P38 ± 9.7; DE, *n* = 6, P40 ± 9) before the onset of high-frequency hearing loss in C57Bl/6 mice (Zheng et al., 1999). Mice were split into two groups, and either placed in a dark room for 7 d (DE) or left in a normal 12/12 h light/dark cycle (NR; Table 1). All animal procedures were approved by the University of Maryland’s Animal Care and Use Committee.

**Table 1.** Animals, fields, and cell numbers imaged

Group	Layer	Animals	Fields	Mean depth	Total cells	Responding cells

Control	L2/3	8	15	189 ± 4μm	1573	989
	L4	9	14	370 ± 11 μm	1202	846
DE	L2/3	6	19	191 ± 4 μm	1919	682
	L4	6	14	354 ± 41 μm	1099	710

Responding cells denote neurons from the total cell population that showed a significant tone-evoked response to at least one frequency (ANOVA across 10 repetitions, *p* < 0.001).

### Cranial window procedure

Mice were initially anesthetized with 4% isoflurane (Fluriso, VetOne) using a calibrated vaporizer (Matrix VIP 3000), which was reduced to 2–2.5% for the craniotomy procedure to maintain stable anesthesia. Body temperature was maintained near 37**°**C with a heating block. Tissue overlying the left auditory cortex was exposed and the skull was affixed to a custom titanium headplate using cyanoacrylate glue (Loctite Prism 454). A small circular craniotomy (3–4 mm in diameter) was performed to expose the surface of the auditory cortex, as determined by skull and vascular landmarks (Stiebler et al., 1997; Dorr et al., 2007). A circular glass coverslip (5 mm, #0 thickness, Warner Instruments) was fixed to the surface of the craniotomy with 1.5–2% warm agarose (Sigma-Aldrich) to dampen pulsations, and was secured with glue on the outer edges to the headplate. For the DE group, mice were maintained in darkness during transfer to the surgery room and anesthesia induction and visualized under infrared illumination. Before beginning the cranial window procedure, mice from both control and DE groups had their eyes sealed shut and covered with black tape, as in our prior experiments (Petrus et al., 2014).

### Two-photon Ca^2+^ imaging

Imaging was largely performed as described previously (Winkowski and Kanold, 2013). Body temperature was maintained at 37**°**C using a homeothermic blanket system (Harvard Apparatus) and a flexible probe to monitor internal temperature. Isoflurane levels were maintained at 1–1.5% for the duration of the imaging session. To prevent cortical cooling and avoid network cortical dysregulation, a constant perfusion of warmed saline (35–37°C) was allowed to flow over the cover-slipped surface of the craniotomy (Kalmbach and Waters, 2012).

Imaging was performed using a two-photon microscope (Ultima, Prairie Technologies) and a MaiTai DeepSee laser (Spectra-Physics), equipped with a GaAsP photo detector module (Hamamatsu) and resonant scanners enabling high-resolution scanning at 30–60 Hz per frame. Excitation was set at 900 nm and focused at 180–200 μm beneath the pia for supragranular L2/3 and 300–400 μm for thalamorecipient L4 imaging. Regions within A1 were scanned at 30 Hz (∼300 × 300 μm) through a 20×, 0.95 NA water-immersion objective (Olympus), with image resolution at 0.58 μm/pixel. Since ACX organization is fairly stereotypical in inbred mice (Stiebler et al., 1997; Liu et al., 2019), we targeted mid-frequency regions based on vascular landmarks.

### Auditory stimulation

Sound stimuli were generated in MATLAB using custom software, presented, and attenuated using Tucker-Davis Technologies RX6, ED1 (Electrostatic Speaker Driver), and PA5 (Programmable Attenuator), and delivered with a free field TDT ES1 speaker placed close to the contralateral (right) ear. Sound intensity was calibrated with a microphone (Brüel & Kjær 4944-A). Sounds were played at 60-dB SPL (∼ 30 dB above mouse hearing threshold for C57BL/6J mice (Zheng et al., 1999), the background strain for the Thy1-GCaMP6s strain used). Auditory stimuli consist of 400-ms-long sinusoidal amplitude-modulated (SAM) tones (5-Hz modulation, cosine phase), ranging from 4 to 64 kHz at quarter octave spacing (spanning four octaves). Each of these 17 stimuli was repeated 10 times with a 6- to 10-s interstimulus interval, for a total of 170 iterations. For each stimulus iteration, a sequence of 100 images were acquired for a duration of 3.3 s, with sound onset at 1.5 s (or at about the 45th frame).

### Data analysis

For image analysis, image sequences were first loaded into ImageJ (NIH) to visually examine whether fluorescent responses were present and whether there were any artifacts from brain motion. Rigid motion correction was performed on image sequences using the ImageJ TurboReg plug-in. Raw fluorescence signals (*F*) of auditory neurons were directly used to calculate frequency time course traces. Cells were manually selected as ring-like regions of interest (ROIs) that cover soma but exclude cell nuclei, and pixel intensity within each ROI was averaged to generate fluorescence over time. Neuropil correction was performed by selecting a circular region with a radius of 20 μm around the cell, excluding all pixels that are contained within other ROIs. For each neuropil mask, the brightest 20% of pixels were also excluded as they might be neural processes from adjacent cells that are also tonally tuned, which otherwise will bias cell response to a smaller value or introduce irregularities in response patterns (Peron et al., 2015). The average fluorescence of this area (background fluorescence, *F_B_*) was then subtracted from the cell’s fluorescence at each time point. Changes in fluorescence (*ΔF/F*) were calculated as [(F – r*F_B_) – (F_0_ – r*F_B_)]/(F_0_ – r*F_B_) (Kerlin et al., 2010; Chen et al., 2012), where F_0_ is estimated by taking the 5th percentile value of the entire subtracted fluorescence trace (for some cells 10th percentile value is chosen to avoid negative F_0_), and r is the contamination ratio 0.7 (Peron et al., 2015). To identify responsive cells, we compared the fluorescence in the stimulus period to the pre-stimulus period. A responsive cell was defined as a cell that showed an increased fluorescence during the stimulus period significantly above baseline (*p* < 0.001, ANOVA) for at least one of the presented stimuli. Only significantly responding cells were analyzed further. Mean time course traces were generated by averaging fluorescence traces over ten repeats, and frequency-tuning curves were determined by taking the maximum (*ΔF/F*) from the mean time course trace across the frames following sound onset. Best frequency (BF) was then defined as the peak of the frequency-tuning curve (the tone which elicits the maximum *ΔF/F* at 60 dB). Spontaneous activity is measured by variance during baseline frames preceding stimulus onset. To estimate baseline activity, the SD of the ΔF/F values of baseline (pre-stimulus) frames across all stimulus presentation trials was determined for each pixel, then averaged. This yielded a single value of baseline ΔF/F variability for each animal. The values of baseline variability were grouped either according to age or rearing condition and compared. Pairwise signal and noise correlation (NC) were calculated as previously described (Liu et al., 2019). We calculated pairwise correlations for all neurons in the imaged field. In brief, NCs were calculated by taking the individual response to each repeat of a sound stimulus, subtracting out the mean response to that particular stimulus, and measuring the covariance of the concatenated responses from every single trial of different stimuli. When trial number is small for each stimulus, signal correlations (SCs) can be strongly biased by NCs (Rothschild et al., 2010, 2013), and thus to overcome this bias, we calculate corrected SCs based on Rothschild et al. (2010):

$${\text{SC}}_{corrected}\left(i,j\right)≜\frac{cov_{corrected}\left(r_{i},r_{j}\right)}{\sqrt{cov(r_{i},r_{i})\cdot cov(r_{j},r_{j})}}.$$

Unlike Rothschild et al. (2010), in the denominator, we used uncorrected expression for $cov(r_{i},r_{i})$ because in practice, $cov_{corrected}\left(r_{i},r_{i}\right)$ can yield negative values for particular $r_{i}$.

### Cell-attached recordings of action potentials *in vitro*

Cell-attached patch clamp recordings were performed *in vitro* in voltage clamp to simultaneously measure spiking activity and *ΔF/F*. Thalamocortical slices containing primary auditory cortex (A1) were prepared as previously described (Zhao et al., 2009; Meng et al., 2015). The extracellular recording solution consisted of artificial CSF (ACSF) containing the following: 130 mM NaCl, 3 mM KCl, 1.25 mM KH_2_PO_4_, 20 mM NaHCO_3_, 10 mM glucose, 1.3 mM MgSO_4_, and 2.5 mM CaCl_2_ (pH 7.35–7.4, in 95% O_2_–5% CO_2_). Action potentials were recorded extracellularly in loose-seal cell-attached configuration (seal resistance typically 20–30 MOhm) in voltage clamp mode. Borosilicate glass patch pipettes were filled with normal ACSF diluted 10%, and had a tip resistance of ∼3–5 MOhm in the bath. Data were acquired with a Multiclamp 700B patch clamp amplifier (Molecular Devices), low-pass filtered at 3–6 kHz, and digitized at 10 kHz using the MATLAB-based Ephus software (Suter et al., 2010). Action potentials were stimulated either by (1) a bipolar electrode placed in L1 or L2/3 to stimulate the apical dendrites of pyramidal cells (pulse duration 1–5 ms) or (2) gradually increasing the extracellular K^+^ concentration (up to ∼8 mM) until spontaneous action potentials began to occur. Data were analyzed offline using MATLAB.

## Results

**Figure 1.**
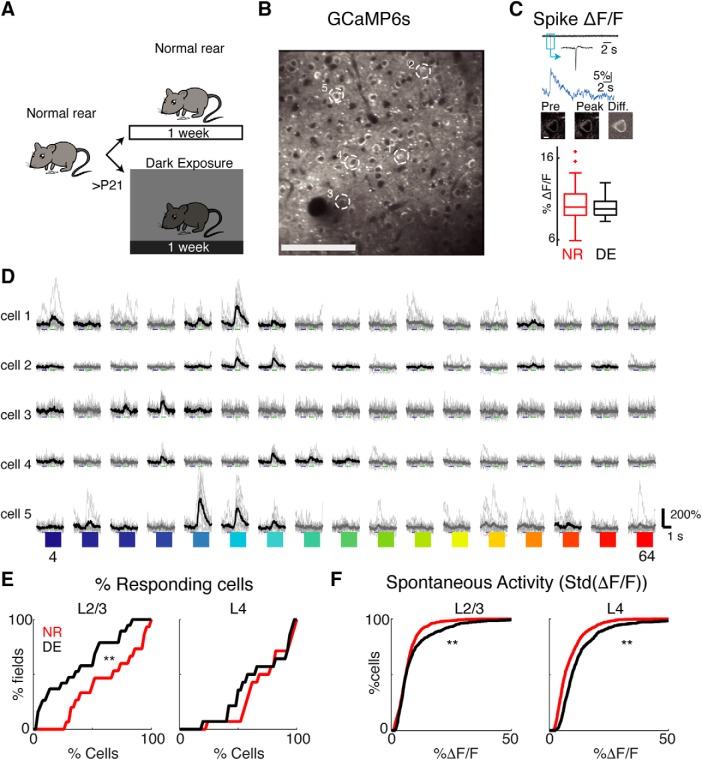
Two-photon Ca^2+^ imaging of GCaMP6s neurons in A1. ***A***, Experimental paradigm. Animals are raised in normal environments until at least P21. Animals then either stay in the normal lighted environment or are DE for 7 d. Cartoon by Zara Kanold-Tso. ***B***, Imaged field with GCaMP6-expressing neurons. Exemplar neurons indicated by white circles. Scale bar = 100 μm. ***C***, Cell-attached patch recordings *in vitro* from GCaMP6S-expressing neurons. Top row shows current trace, with inset showing magnified action potential. Middle row shows the corresponding Ca^2+^ rise (*ΔF/F*) in response to one action potential. Bottom row shows the corresponding two-photon fluorescence images: first image is of cell preceding spike, middle image is of cell at peak of fluorescent response, and third image shows the difference (scale bar = 5 μm). Boxplots shows median and interquartile range of fluorescent-evoked responses to one spike in control and DE mice. DE does not alter the amplitude of spike-induced fluorescence transients (mean ΔF/F ± SEM per spike: NR = 10.25 ± 0.29%, *n* = 62 spikes; DE = 9.97 ± 0.20%, *n* = 37 spikes; two-sample Kolmogorov–Smirnov test, *p* = 0.16). ***D***, Sound evoked fluorescence traces in five exemplar cells (indicated in ***B***). Black lines indicate mean trace for responses that passed the significance criterion (ANOVA *p* < 0.001), while thin gray traces show individual trials. Colors indicate tone frequency 4–64 kHz. ***E***, Fraction of responsive cells decreases in L2/3 following DE (mean ± SD, NR = 64.2 ± 26.9%, DE = 37.4 ± 28.4%, Wilcoxon rank-sum test, *p* = 0.0167) with no change in L4 (NR = 72.1 ± 22.0%, DE = 66.9 ± 25.4%, *p* = 0.35). ***F***, Spontaneous activity, as measured by SD of the baseline in ΔF/F traces, increased in L4 and L2/3 after DE (NR median ± iqr L2/3 = 6.1 ± 4.7, DE L2/3 = 6.2 ± 6.2, *p* = 0.0018; NR L4 = 6.6 ± 6.7, DE L4 = 9.6 ± 7.9; Wilcoxon rank-sum test, *p* < 10^−28^).

We aimed to investigate whether a brief period of visual deprivation (DE) altered the single cell and population responses in L2/3 and L4 in A1 (Fig. 1*A*). To visualize the activity of A1 neurons we used two-photon Ca^2+^ imaging in *Thy1*-GCaMP6s (GP4.3) mice (JAX strain 024275; Dana et al., 2014) that were randomly assigned to DE (*n* = 6) or NR (*n* = 9; Table 1) groups (NR P38 ± 9.7; DE P40 ± 9). Ca^2+^ imaging allowed us to measure responses from hundreds of neurons in each layer (Table 1). Since Ca^2+^ indirectly reports neuronal activity, we first tested whether DE altered the relation of spiking activity to cellular Ca^2+^ dynamics. Prior *in vitro* studies indicated that DE does not cause changes in intrinsic spiking properties of L4 and L2/3 cells (Meng et al., 2015, 2017a). We performed cell-attached patch clamp recordings *in vitro* (in voltage clamp) to simultaneously measure spiking activity and *ΔF/F*. The recordings showed that DE did not alter the amplitude of spike-induced fluorescence transients (Fig. 1*C*). Together with the fact that DE did not cause changes in intrinsic spiking properties of L4 and L2/3 cells (Meng et al., 2015, 2017a), these data suggest that DE did not change the intrinsic properties and Ca^2+^ dynamics of A1 neurons.

### DE increases the amplitude of sound-evoked responses in L4 and frequency selectivity of single neurons in both L2/3 and L4

To characterize the single-cell response properties of neurons in control and DE mice, we imaged ∼300 × 300 μm regions within L2/3 and L4 and presented pure tones (4–64 kHz, 60 dB; Fig. 1*D*). We first identified cells that responded to these tonal stimuli. A cell was classified as responsive if it responded significantly to at least one of the presented stimuli. After DE, the fraction of tonally responsive cells in L4 did not change, however, in L2/3 fewer cells responded to these tonal stimuli (Fig. 1*E*) indicating a sparsification of cortical responses in supragranular layers.

Single unit microelectrode recordings have shown that L4 cells in DE animals have higher spontaneous and peak evoked firing rates (Petrus et al., 2014). We thus investigated whether these changes on the single cell level after DE were also present in L2/3. To evaluate the spontaneous activity of GCaMP6s-expressing neurons, we measured the fluorescence transients preceding the onset of the stimulus and during long-duration imaging without any stimulus presentation. We characterized the spontaneous activity as the SD of the fluorescence trace. The spontaneous activity increased in both L4 and L2/3 after DE (Fig. 1*F*). Thus, DE increased spontaneous activity in both L4 and L2/3 and this increase in spontaneous activity is unlikely to underlie the decreased responsiveness in L2/3.

**Figure 2.**
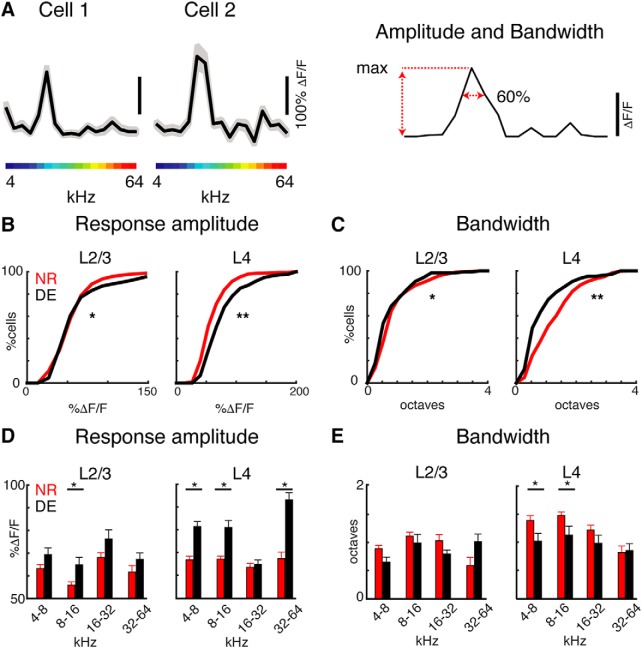
DE increases the responsiveness and frequency selectivity of neurons in both L4 and L2/3. ***A***, Exemplar tuning curves (mean ± 1.96*SEM) of two cells obtained from tone evoked responses. ***B***, ***C***, Cumulative distribution functions of response amplitudes (***B***) and bandwidth. ***B***, Response amplitude measured by peak ΔF/F increased in L4 and L2/3 after DE [mean ± SEM, L4 NR = 65.9 ± 0.9%, DE = 83.0 ± 1.4%; Kolmogorov–Smirnov (KS) test, *p* < 10^−16^; L2/3 NR = 61.3 ± 1.0%, DE = 67.35 ± 1.6%; KS test, *p* = 0.027]. ***C***, Bandwidth decreased in L4 and L2/3 after DE (mean ± SEM, L4 NR = 1.17 ± 1.14, DE = 0.68 ± 0.79 octaves; KS test, *p* < 10^−5^; L2/3: NR = 0.98 ± 0.04, DE = 0.86 ± 0.06, KS test; *p* = 0.01). ***D***, ***E***, Response amplitudes (***D***) and bandwidth (***E***) in octave frequency bins. ***E***, Response amplitudes in L2/3 were increased for cells with BFs of 8–16 kHz (4–8 kHz *p* = 0.066; 8–16 kHz *p* = 0.004; 16–32 kHz *p* = 0.07; 32–64 kHz *p* = 0.32). Response amplitudes in L4 were increased for cells with BFs of 4–8, 8–16, and 32–64 kHz (4–8 kHz *p* = 5.2 × 10^−8^; 8–16 kHz *p* = 2.4 × 10^−6^; 16–32 kHz *p* = 0.7; 32–64 kHz *p* = 1.4 × 10^−5^). ***D***, Bandwidth in L2/3 was similar in each bin (4–8 kHz *p* = 0.073; 8–16 kHz *p* = 0.45; 16–32 kHz *p* = 0.075; 32–64 kHz *p* = 0.089). Bandwidth in L4 was decreased for cells with BFs of 8–16 and 16–32 kHz (4–8 kHz *p* = 0.039; 8–16 kHz *p* = 0.029; 16–32 kHz *p* = 0.29; 32–64 kHz *p* = 0.86).

The decrease in tonal responsiveness could be due to altered frequency tuning of A1 neurons. Prior microelectrode recordings have shown that L4 cells in DE animals have increased frequency selectivity (Petrus et al., 2014) and since L2/3 receives input from L4 (Meng et al., 2015, 2017a) such changes could also be present in L2/3. We thus generated tuning curves for each responding cell based on the maximum evoked response during tone presentation (Fig. 2*A*). We first measured the amplitude of the evoked responses at the BF. The amplitude of the evoked responses after DE was increased in both layers, but the magnitude of change was larger in L4 (Fig. 2*B*). This is consistent with electrophysiological recordings and the strengthening of thalamocortical afferents to L4 (Petrus et al., 2014). We next evaluated the frequency selectivity of cells in DE and NR mice by calculating the bandwidth of the tuning curves. We measured the normalized bandwidth using a peak-related threshold (BW_60%_) to characterize changes in tonal receptive fields. We find that the bandwidth was decreased in both L4 and L2/3 cells after DE as compared to cells from NR animals (Fig. 2*C*) with a greater magnitude of decrease in L4 than L2/3. Together, these results show that on the single cell level, changes after DE are similar in both L4 and L2/3 with the exception that response amplitudes in L4 but not L2/3 increase after DE.

Thus, DE after the critical period can alter the sound evoked responses of A1 neurons in both L4 and L2/3 but the magnitude of changes might be greater in L4 than L2/3. Together, these results indicate that while fewer cells responded to tones after DE, those cells that did respond to tones in A1 become more responsive and selective to sound after DE in both L4 and L2/3. These *in vivo* imaging results are consistent with prior microelectrode recordings in L4 (Petrus et al., 2014) and extend those prior observations to L2/3.

### DE alters the distribution of frequency selectivity in A1

**Figure 3.**
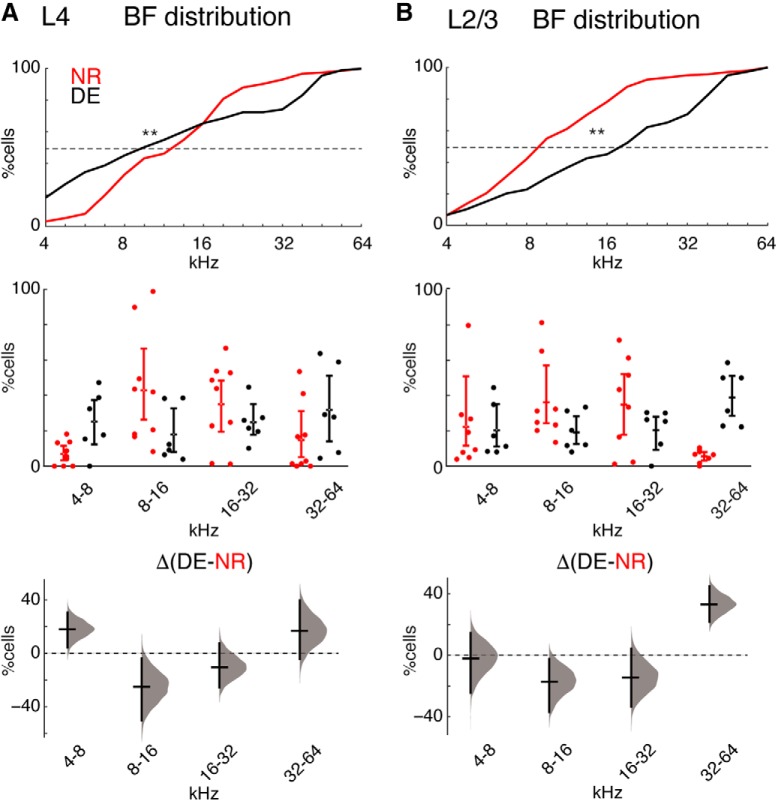
DE alters the representation of sound frequencies in A1. Distribution of BFs in NR and DE in all imaging fields from L4 (***A***) and L2/3 (***B***) and across mice (Table 1). ***A***, top panel, Cumulative distributions showing the spread of BFs in NR (red) and DE (black) in imaging fields from L4. The BF distribution of cells differs between DE and NR [Kolmogorov–Smirnov (KS) test; L4 *p* < 10^−23^]. Lower panels, Same data as in top panel by animals (nine mice NR; six mice DE) and binned into octaves. The mean differences for the comparisons are shown by Cumming estimation plot. The raw data are plotted on the upper axes; summary measurements (mean ± SD) are shown as lines. Mean differences for each frequency bin are plotted on the lower plot as a bootstrap sampling distribution (DABEST). Mean differences are depicted as horizontal lines; 95% confidence intervals are indicated by the ends of the vertical error bars (4–8 kHz: 18.2% [95.0%CI, 4.34, 30.7], *p* = 0.0432 Mann–Whitney; 8–16 kHz: –24.9% [95.0%CI –50.2, –3.86], *p* = 0.0518; 16–32 kHz: –10.2% [95.0%CI, –25.5, 7.41], *p* = 0.377; 32–64 kHz: 17% [95.0%CI, –4.21, 39.8], *p* = 0.0872). Effect size [CI width, lower bound, upper bound]. ***B***, top panel, Cumulative distributions showing the spread of BFs in NR (red) and DE (black) in imaging fields from L2/3. The BF distribution of cells differs between DE and NR (KS test; L2/3 *p* < 10^−40^). Lower panels, Same data as in top panel by animals (eight mice NR; six mice DE) and binned into octaves. The mean differences for the comparisons are shown by Cumming estimation plot. Mean differences are depicted as in ***A*** (4–8 kHz: 1.8% [95.0%CI, –24.3, 14.4], *p* = 0.651 Mann–Whitney; 8–16 kHz: –17.1% [95.0%CI, –26.9, 2.41], *p* = 0.175; 16–32 kHz: –14.3% [95.0%CI, –33.3, 4.33], *p* = 0.22; 32–64 kHz: 32.28% [95.0%CI, 21.68, 44.99], *p* = 0.0024).

Our results indicate that tone-responsive cells in L4 of A1 showed higher response amplitude and cells in both layers of A1 showed increased selectivity to sound after DE. However, these changes on the single cell level do not explain why fewer responsive neurons exist in L2/3 after DE. Neurons in sensory cortices can adjust their tuning based on behavioral demands (Fritz et al., 2003, 2005; Polley et al., 2006; Winkowski et al., 2013; Francis et al., 2018). Moreover, early sensory experience can alter the amount of A1 territory that responds to tones of a certain frequency (Zhang et al., 2001). We thus reasoned that it might be possible that as a population, cells shifted their stimulus preference. To explore the possibility of such a scenario, we investigated the distribution of preferred frequencies in NR and DE mice over the population of imaged cells. In microelectrode studies of A1 plasticity A1 is sparsely sampled, tessellated, and the relative areas of regions with certain BFs is calculated (Zhang et al., 2001). Since *in vivo* cellular imaging revealed that neighboring cells can show very different BFs (Bandyopadhyay et al., 2010; Rothschild et al., 2010; Winkowski and Kanold, 2013; Maor et al., 2016), we do not calculate the fractional A1 area but instead the fraction of A1 cells showing a certain BF. In NR animals, cells in both L4 and L2/3 showed preferred frequencies ranging from 4 to 64 kHz with most neurons preferring tones between 8 and 32 kHz (Fig. 3), consistent with the most sensitive area of mouse hearing and the overrepresentation of such frequencies in A1 (Stiebler et al., 1997; Guo et al., 2012; Liu et al., 2019). In contrast, DE mice showed an altered distribution of BFs with relatively more cells responding to high frequencies (32–64 kHz; Fig. 3*B*). Across animals, we observed an increase in the proportion of cells selective for high frequencies (32–64 kHz) in L2/3 and an increase in cells selective for low frequencies (4–8 kHz) combined with decrease for mid-frequencies (8–16 kHz) in L4 (Fig. 3*A*). Together, these results suggested that the functional representation of tones in A1 broadens after DE.

### DE decreases the pairwise activity correlations between neurons

Sensory stimuli are not only encoded by single neurons but by populations of neurons, and activity correlations between neurons contribute to information encoding (Averbeck et al., 2006). In both L4 and L2/3, nearby cells show high SCs, which reflect stimulus-driven correlated activity, and NCs, which represent stimulus-independent, trial-to-trial covariance (Winkowski and Kanold, 2013). Pairwise correlations can serve as a proxy for functional connections with interconnected cells having increased NCs. Since our circuit analysis in DE animals showed a refinement of functional interlaminar and intralaminar connections (Meng et al., 2015, 2017a) we reasoned that pairwise correlations might decrease. We thus examined whether DE altered the level of correlated activity between neurons in L4 and L2/3 by calculating the pairwise correlation of neurons in the imaged field.

**Figure 4.**
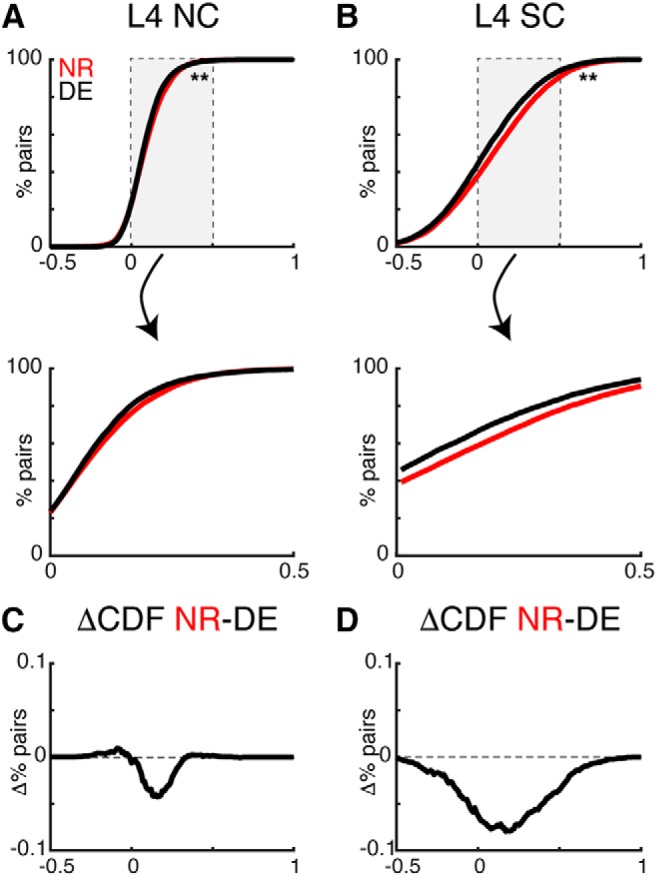
DE decreases pairwise activity correlations in L4. ***A***, ***B***, CDFs of pairwise noise (NC) and signal correlations (SC) in L4 from NR and DE animals [Kolmogorov–Smirnov (KS) test; L4 NC *p* = 3.7 × 10^−4^; L4 SC *p* = 6.5 × 10^−28^]. Lower panels show magnified view of center of distributions. ***C***, ***D***, Differences between the CDFs show a broad decrease in SC in L4.

**Figure 5.**
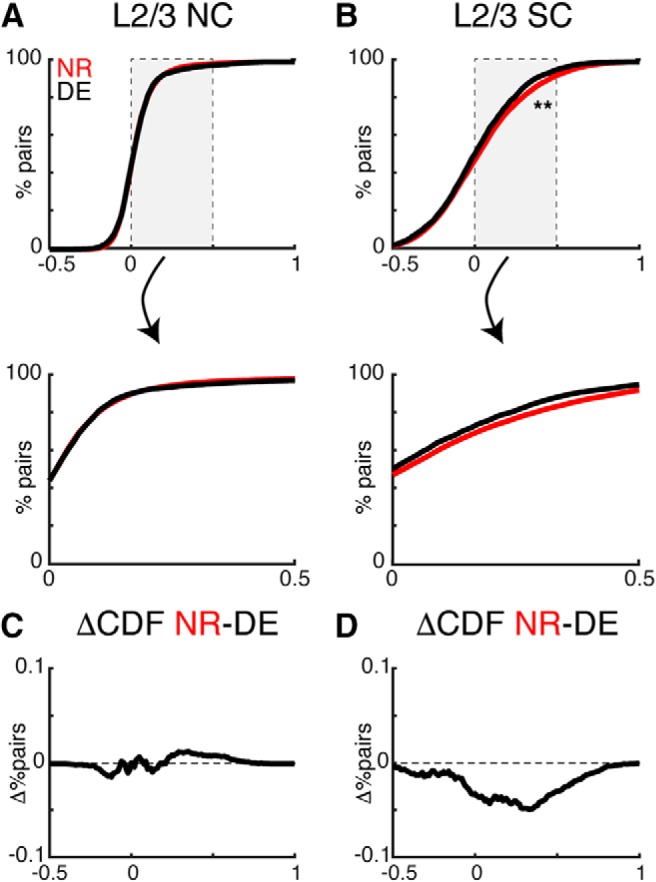
DE decreases pairwise activity correlations in L2/3. ***A***, ***B***, CDFs of pairwise noise (NC) and signal correlations (SC) in L2/3 from NR and DE [Kolmogorov–Smirnov (KS) test; L2/3 NC *p* = 0.37; L2/3 SC *p* = 7.7 × 10^−9^]. Lower panels show magnified view of the center of distributions. ***C***, ***D***, Differences between the CDFs show a broad decrease in SCs in L2/3.

DE resulted in a decrease in both NC and SC between simultaneously imaged L4 cells (Fig. 4). However, in contrast to the effects in L4 NCs were largely unchanged in L2/3 (Fig. 5*A*). DE resulted in a decrease in SC between simultaneously imaged L2/3 cells (Fig. 5*B*). Together, these results show that DE not only alters the tuning of single neurons in L4 and L2/3 but also the local activity relationships between neurons. The decreased pairwise correlations indicate a sparsification of the population activity in A1 consequent to DE.

**Figure 6.**
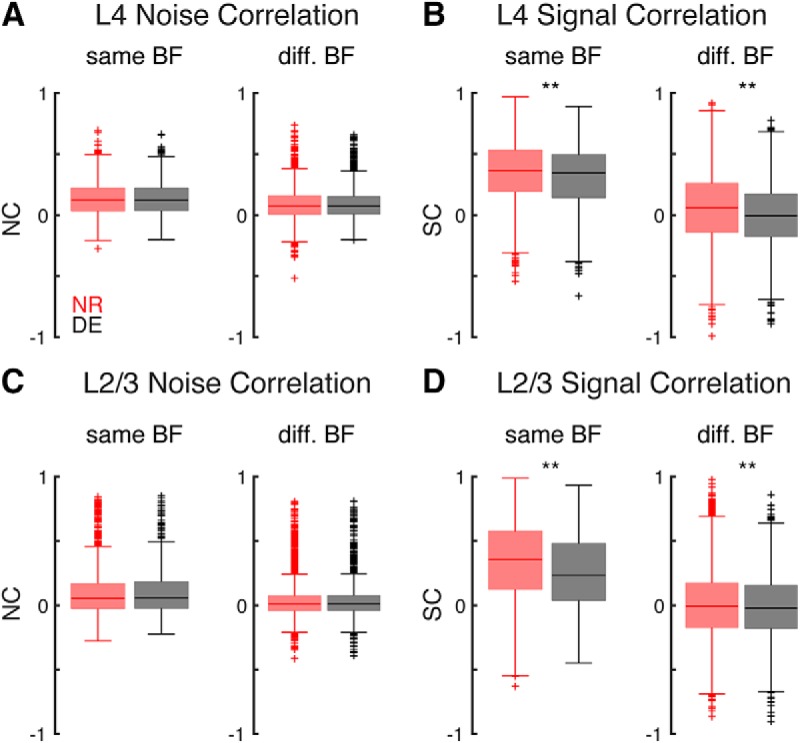
DE decreases pairwise SCs for both co-tuned and non-co-tuned neurons. ***A***, L4 NCs in NR and DE for cells with similar and different BF (*p* = 0.95; *p* = 0.97). ***B***, L4 SCs in NR and DE for cells with similar and different BF (*p* < 0.0036; *p* = 5.4 × 10^−35^). ***C***, L2/3 NCs in NR and DE for cells with similar and different BF (*p* = 0.37; *p* = 0.96). ***D***, L2/3 SCs in NR and DE for cells with similar and different BF (*p* < 1.2 × 10^−5^; *p* < 0.0094).

The changes in SCs and NCs after DE might differ depending on the BF relationships of the cell pair. We found that L4 NCs did not change after DE for cell pairs that were co-tuned and for cell pairs with different BFs (Fig. 6*A*). L4 SCs were higher between co-tuned cells and the decrease in L4 SC after DE occurred for both co-tuned and non-co-tuned cells (Fig. 6*B*). We observed similar changes in L2/3 (Fig. 6*C*,*D*). These results indicate that the sparsification of responses consequent to DE did not depend on the tuning relationship of neuronal pairs.

**Figure 7.**
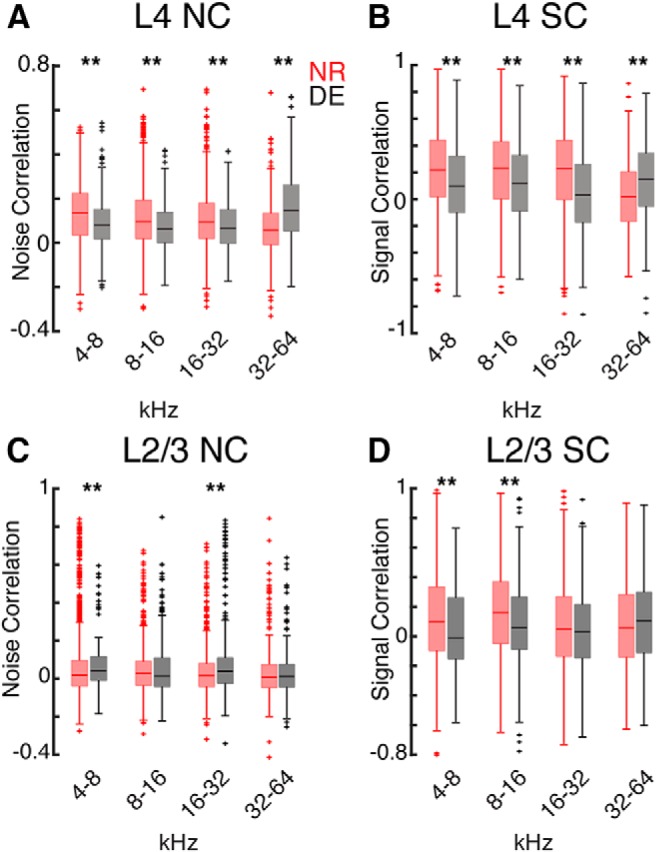
DE causes frequency specific effects on SCs and NCs. ***A***, Boxplots showing NCs for L4 cell pairs with BFs of 4–8, 8–16, 16–32, and 32–64 kHz from NR and DE (*p* = 7.3 × 10^−18^, *p* = 1.2 × 10^−10^, *p* = 8.3 × 10^−5^, *p* = 1.9 × 10^−22^). ***B***, Boxplots showing SCs for L4 cell pairs with BFs of 4–8, 8–16, 16–32, and 32–64 kHz from NR and DE (*p* = 7.5 × 10^−17^, *p* = 4.4 × 10^−12^, *p* = 5.4 × 10^−20^, *p* = 8.38 × 10^−10^). ***C***, Boxplots showing NCs for L2/3 cell pairs with BFs of 4–8, 8–16, 16–32, and 32–64 kHz from NR and DE (*p* = 0.006, *p* = 0.48, *p* = 0.0004, *p* = 0.73). ***D***, Boxplots showing SCs for L2/3 cell pairs with BFs of 4–8, 8–16, 16–32, and 32–64 kHz from NR and DE (*p* = 0.002, *p* = 0.0001, *p* = 0.22, *p* = 0.16).

We next investigated whether changes in pairwise correlations were similar for cell pairs across the hearing range. We thus separately calculated pairwise correlations between cells with frequency preference in different octave bands (Fig. 7). In L4 DE decreased NCs and SCs for pairs of cells in the 4- to 8-, 8- to 16-, and 16- to 32-kHz frequency groups but increased for cell pairs between 32 and 64 kHz (Fig. 7*A*,*B*). In L2/3, DE increased NCs for pairs in 4- to 8- and 16- to 32-kHz range, while SCs decreased for cells in 4- to 8- and 8- to 16-kHz band (Fig. 7*C*,*D*). These results indicate that DE increases the number of cells responding to high frequencies and in addition also increases the pairwise correlations between L4 cells in high frequency bands while decreasing correlations elsewhere.

**Figure 8.**
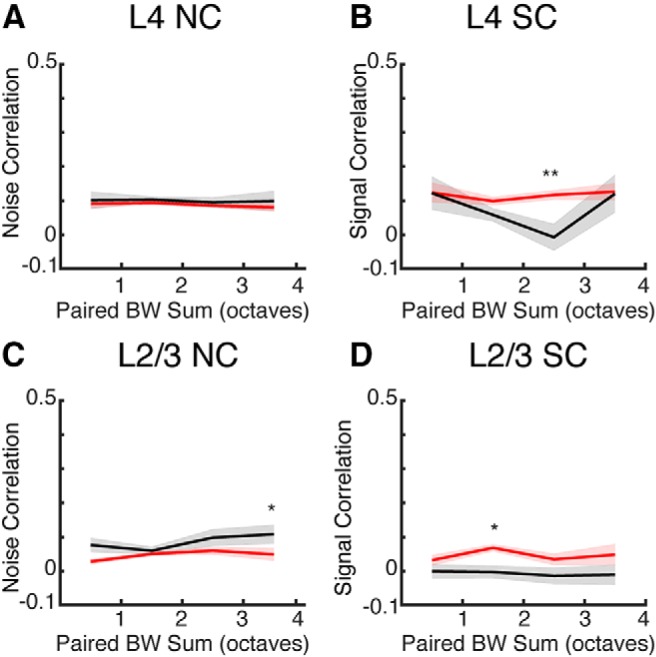
DE-induced changes in pairwise correlations can depend on bandwidth sum. ***A***, NCs for L4 cell pairs as a function of summed bandwidth from NR and DE. ***B***, SCs for L4 cell pairs as a function of summed bandwidth from NR and DE from NR and DE (** indicates significant difference at *p* < 0.01). ***C***, NCs for L2/3 cell pairs as a function of summed bandwidth from NR and DE (* indicates significant difference at *p* < 0.05). ***D***, SCs for L2/3 cell pairs as a function of summed bandwidth from NR and DE (* indicates significant difference at *p* < 0.05).

SCs are a measure of similarity between tuning curves of neurons, thus changes in SC could depend on the bandwidths of the neurons in a pair. For each cell pair we summed the bandwidth of each neuron and plotted the NC and SC as a function of bandwidth sum in octaves (Fig. 8*A*,*B*). There was no dependence of NC and SC on bandwidth sum in NR mice. After DE cell pairs with intermediate bandwidth sum in both L4 and L2/3 showed decreased SC (Fig. 8*B*,*D*) indicating that DE causes reduced SCs for neuronal pairs with intermediate or mismatched bandwidths (either one narrow and one broad or both moderate). In L2/3 neuronal pairs, NCs increased for pairs with broad bandwidth sums (Fig. 8*C*). Together, these results indicate that DE had the largest effect on SCs between neurons with intermediate combined bandwidth. This suggests that the change in correlations after DE is not solely due to changes in bandwidth but also due to changes in the relative tuning by cells in the imaged fields.

## Discussion

Using *in vivo* two-photon Ca^2+^ imaging we show that brief (7 d) periods of visual deprivation in adult mice resulted in robust cross-modal changes in population activity of thalamorecipient and supragranular layers of A1.

On the single cell level, we find that DE after the critical period can alter both spontaneous and sound-evoked responses of A1 neurons in both L4 and L2/3. Specifically, after DE, cells show increased spontaneous activity in both layers, increased responsive amplitudes and decreased bandwidth in both L4 and L2/3. These changes after DE in L4 are consistent with prior microelectrode studies of L4 (Petrus et al., 2014) and we here extend those findings to L2/3. Increase in spontaneous activity is reminiscent of changes seen after within modality sensory deprivation, which seems to correlate with engagement of cortical plasticity. For example, spontaneous firing rate is increased in A1 following noise-induce hearing loss (Komiya and Eggermont, 2000; Seki and Eggermont, 2003) and in V1 after a few days of DE (Bridi et al., 2018). In the case of within modality sensory deprivation, it is likely driven by a decrease in feedforward inhibition by loss of sensory drive, as well as a decrease in inhibitory synaptic function (Gao et al., 2014, 2017). However, this is unlikely to be the case for cross-modal sensory deprivation. To the contrary, we reported that inhibitory synaptic transmission increases in A1 L4 and L2/3 following DE (Petrus et al., 2015). This is similar to increased inhibition following cross-modal rewiring of A1 (Mao and Pallas, 2013). It is plausible that cross-modal deprivation may lead to temporary disinhibition similar to what is describe for within modality sensory deprivation (Kuhlman et al., 2013), which is thought to enable plasticity. However, at a later time point inhibition may be increased to consolidate the changes. We previously found that DE reduces neuronal thresholds (Petrus et al., 2014). Since we used a fixed sound level, cells in DE were tested at a relative sound level that was slightly higher compared to threshold than in cells in NR. As bandwidth increases with sound level for most auditory neurons, our results might underestimate the decrease in bandwidth after DE.

Our results corroborate that increased spontaneous activity correlates with cortical plasticity. *In vivo* cross-modal changes on the single cell level are also consistent with prior *in vitro* studies which have shown extensive synaptic and circuit level changes in both L4 and L2/3 (Petrus et al., 2014, 2015; Meng et al., 2015, 2017a). However, our results further indicate that changes in sound-evoked properties are larger in L4 than L2/3, which suggests that previously observed potentiation of TC synapses (Petrus et al., 2014) and recurrent L4 excitatory inputs (Petrus et al., 2015) may play a prominent role in sculpting A1 functionality. While it is conceivable that anesthesia obscures even more extensive circuit changes in L2/3, we speculate that since the pure-tone bandwidth of L2/3 cells is already narrower than that of L4 neurons (Winkowski and Kanold, 2013), DE might alter other features of the cells’ receptive field which are not revealed with pure tones such as spectral contrast sensitivity (Barbour and Wang, 2003). Furthermore, synaptic circuit changes in A1 L2/3 are rather complex compared to those seen in L4. For example, in addition to potentiation of feedforward synapses from L4, there is large scale depression of lateral inputs within L2/3 (Petrus et al., 2015), as well as refinement of both inputs (Meng et al., 2015). Hence functional consequence of DE on A1 L2/3 function is difficult to predict. Regardless, our results suggest that DE induced functional adaptation of A1 L2/3 circuits may be geared toward expanding representation of higher frequency tones and sparsification of population coding.

*In vivo* two-photon Ca^2+^ imaging also allows us to identify population and network level changes after DE. After DE, we observe a decrease in pairwise correlations between neurons in both L4 and L2/3 but a selective increase for pairs of cells tuned to 32–64 kHz. Since SCs are reflective of stimulus-carrying inputs and since thalamocortical inputs drive L4 neurons, our observed decrease in L4 SCs suggests a refinement of thalamocortical input to L4 neurons after DE. This would be consistent with the observation of decreased bandwidth and increase responses of L4 neurons after DE (Petrus et al., 2014). Since *in vitro* data showed a strengthening of thalamocortical synapses (Petrus et al., 2014) our results suggest that DE after the critical period leads to both a refinement and strengthening of thalamocortical synapses. On the other hand, NCs can be reflective of intracortical connections. The observed decrease in NC in L4 after DE is consistent with changes in interlaminar connections to L4 (Meng et al., 2017a). In L2/3 we also observe overall decreased SCs, suggesting that ascending connections from L4 refine consistent with *in vitro* observations (Meng et al., 2015) and also with increased mIPSCs frequency (Petrus et al., 2015) indicating increased inhibitory tone. We did not find changes in L2/3 NC except for small increases in 4- to 8- and 16- to 32-kHz pairs. NCs likely reflect the extensive intralaminar connectivity of L2/3 neurons (Atzori et al., 2001; Levy and Reyes, 2012; Meng et al., 2017b), thus the lack of consistent change in NCs would suggest no changes in intralaminar connectivity. However, prior laser-scanning photostimulation (LSPS) studies suggested that intra-L2/3 circuits refine following DE (Meng et al., 2015). The difference might be due to the limited spatial resolution of LSPS, which does not allow investigation of connections within the 100-μm range that was included in our current study.

After DE, single neurons show higher spontaneous and sound-evoked responses in both layers. However, this increase in firing rates did not cause a general increase in pairwise activity correlations except for pairs in the 32- to 64-kHz band. This suggests that activity correlations can be independently controlled, possibly via selective engagement of inhibitory circuits. Indeed, both excitatory and inhibitory circuits to L4 neurons change after DE (Petrus et al., 2015; Meng et al., 2017a). Changes in activity correlations are also seen after noise exposure during the critical period and after and changes in inhibition might underlie this change (Zhang et al., 2002; Zhou and Merzenich, 2012).

Feedforward projections determine the initial tuning preference of neurons and maintain tonotopy on a coarse scale, whereas intracortical inputs can either broaden spectral tuning via excitatory synapses, or sharpen receptive fields via inhibitory inputs (Wehr and Zador, 2003; Kaur et al., 2004, 2005; Tan et al., 2004; Wu et al., 2008; Happel et al., 2010). Feed-forward projections from the medial geniculate body (MGB) of the thalamus determine the frequency preference of L4 neurons (Li et al., 2013). We here observed a decrease in SCs in L4. The decrease in SCs in L4 suggests that after DE, MGB inputs to L4 cells are refined, either by changing the sets of frequency inputs or the strength of these inputs to neighboring L4 cells. Alternatively, intracortical inputs to L4 neurons could have changed. Indeed, *in vitro* circuit mapping studies have shown that L2/3-L4 connections but not L4-L4 connections refine after DE (Meng et al., 2017a). Thus, feedback projections from L2/3 could contribute to the adjustment of the spatial representation of sound frequency in L4.

While we observed a decrease in SCs in both L4 and L2/3, the magnitude of these changes was larger in L4 than L2/3. This could indicate a ceiling effect signifying a minimum in SCs because SCs are larger in L4 than L2/3 (Winkowski and Kanold, 2013). Alternatively, these results could indicate that a certain level of population response sparsification is maintained. Since L2/3 receives its dominant ascending input from L4 and since intracortical circuits to both L4 and L2/3 change after DE (Meng et al., 2015, 2017a; Petrus et al., 2015), this could suggest that L4-L2/3 circuit refinement after DE might normalize the frequency representation.

Our results also show that DE induces a decorrelation of the sound evoked population activity in A1. Decorrelation of sensory responses can lead to increased encoding fidelity of represented stimuli (Averbeck et al., 2006) and our prior studies of A1 have shown that decorrelation can improve discrimination performance (Winkowski et al., 2013) and that engagement in tone detection tasks can lead to temporary decorrelation of A1 responses during trials (Francis et al., 2018). Thus, we speculate that a period of DE in which animals rely on sound and not vision might cement these temporary changes.

After DE, a lower fraction of neurons in both layers are selective for mid-frequencies and a relatively higher fraction of cells are selective for high-frequencies. While these changes could be due to a sampling bias in our experiments, we sampled many fields across A1 (Table 1) making such systematic differences unlikely. Rather, it is more probable that DE has changed the representation of sound in A1. Moreover, we find that while overall SCs decreased after DE, pairs within the 32- to 64-kHz frequency range increased their SCs indicating a differential effect on low-frequency and high-frequency cells. The increase in SC for cells in the 32- to 64-kHz range points to a selective effect of DE on high-frequency cells. A1 neurons can adapt to stimulus statistics by decreasing responsiveness to frequently occurring sounds (Ulanovsky et al., 2003; Nelken, 2004, 2014; Winkowski et al., 2013; Pérez-González and Malmierca, 2014). We speculate that these changes reflect, and are dependent on, the sound experience of the animal during DE. While we have previously not detected any differences in the sound environment in NR and DE, we did find that animal vocalizations under our conditions are most frequent in the mid-frequency range (Petrus et al., 2014). However, besides vocalizations, animals are exposed to a variety of other ambient or self-generated sounds (e.g., from locomotion). While ambient sound level was similar in DE and NR, we did not compare the ambient sound spectrum during the housing period nor did we observe the behavior of the animals during NR and DE. Thus, it is possible that other sounds besides vocalization differed between NR and DE.

Despite the caveats, we speculate that DE amplifies adaptive processes leading to changes in synaptic strength that reflect the sound environment, consistent with our observation of decorrelation of responses. It is tempting to conjecture that pairing the presence of specific sound stimuli with DE might be able to drive targeted changes in A1. Consistent with this idea, cross-modal sensory deprivation has been shown to facilitate LTP in V1 (Rodríguez et al., 2018).

Recent studies revealed that A1 responses sparsify with development and that auditory experience early in life can shape this process (Liang et al., 2019; Meng et al., 2019). We here show that visual experience later in life has a similar capability. Our results reveal changes in the population activity and frequency organization of A1 well after the critical period, suggesting that cross-modal plasticity of A1 circuits may underlie the improvements in auditory perception in humans following vision loss (Lessard et al., 1998; Röder et al., 1999; Gougoux et al., 2004; Voss et al., 2004). These results add to the mounting evidence that experience-dependent plasticity is not restricted to early developmental windows, and that cross-modal sensory experience has the power to alter network circuitry and population dynamics even into adulthood. Thus, it might be possible to harness this environmental manipulation to restore function lost as a result of impaired developmental experiences, and consistent with this, a brief duration of auditory or whisker deprivation is able to restore ocular dominance plasticity in adult V1 (Rodríguez et al., 2018; Teichert et al., 2018, 2019).

## Synthesis

Reviewing Editor: Siegrid Löwel, University of Goettingen Decisions are customarily a result of the Reviewing Editor and the peer reviewers coming together and discussing their recommendations until a consensus is reached. When revisions are invited, a fact-based synthesis statement explaining their decision and outlining what is needed to prepare a revision will be listed below. The following reviewer(s) agreed to reveal their identity: NONE. Note: If this manuscript was transferred from JNeurosci and a decision was made to accept the manuscript without peer review, a brief statement to this effect will instead be what is listed below.

Summary:

In this well written and concise manuscript the authors extend on own previous work investigating crossmodal changes in the auditory cortex after short visual deprivation in adulthood. In general, the study is well designed, carefully conducted and analyzed with appropriate quantitative and statistical means. Using modern imaging techniques to investigate the activity of individual neurons in GCAMP6s expressing transgenic mice the study describes changes in spontaneous activity, response amplitudes and tuning bandwidths in layer 4 and 2/3 which largely replicate their own results obtained in earlier studies employing multielectrode recordings. A novel aspect of the current work is the analysis of concerted activity between neurons within each layer studied. Here, the authors report reduced correlated spontaneous and stimulus driven activity in layer 4 and decreased correlation of stimulus driven activity in layer 2/3 after visual deprivation. These latter findings represent the main novelty of the paper and provide additional insights into the mechanisms and changes during crossmodal plasticity in adulthood.

Major points:

As currently the study mostly corroborates findings from the senior author's labs the paper would substantially benefit from an extended analysis of the exciting new aspects namely the correlations between neurons. For example, signal correlations (SC) and noise correlations (NC) could be correlated with the bandwidth of the cells and then compared between normal reared (NR) and dark exposed (DE) animals. An increased correlation in DE animals could be interpreted as the changes in SC and NC to be driven by bandwidth reduction. This would further strengthen the conclusion of the authors that decrease in SCs in L4 suggests that after DE MGB inputs to L4 have refined. A further possibility could be to split the signal correlations according to BF as the authors suggest that the sparsening of responses could enhance encoding of auditory stimuli. It would further be interesting to look at Off responses as they seem to be correlated over a much larger distance in A1 as shown by a recent landmark study of the authors (Liu et al. 2019).

The authors suggest that the DE leads to stronger reliance on sound which leads to plastic changes based on behavioral needs and that this hypothesis is consistent with the data. However, we are not entirely convinced that such a strong interpretation is validated by the data. Taken this hypothesis into account we would have expected much more contingent changes in the data e.g. changes in response amplitudes as well as bandwidths and correlations in the frequency range of behaviorally relevant sounds. In contrast, the observed changes seem to be rather unsystematically spread across observables, layers and tonotopic frequency. For example, the relative abundance is increased for cells with high BFs (and low for layer 4), but the response amplitude is increased from 8 - 16 kHz, while the bandwidth of tuning is decreased for low BF neurons and so on. The authors also suggest that the lower fraction of mid-frequency selective neurons could be due to the abundance of mid-frequency vocalizations. However, the relevant data in Petrus et al. 2014 (Fig. S5) show that roughly 80 % of vocalizations have peak frequencies below 20 kHz and about 50 % of vocalizations below 10 kHz. Thus if the authors hypothesis is valid one could have rather expected a reduction for low frequencies where an increase in abundance was seen. But still from the cited earlier work it is also difficult to assess or speculate why some BFs are reduced or enhanced in abundance. Maybe the increase at high BFs is due to an increase in peripheral threshold at high frequencies (age-related hearing loss, see below)? What was the auditory background within the vivarium for the animals? Where they housed in the same room but at different times (DE vs. NR?). In the previous work, there is an analysis of ambient sound level and vocalizations but no comparison of ambient sound spectrum. Small differences in these might be sufficient to drive relative changes. Further, there is no mention of the relative frequency of the vocalizations between both groups. The driving force could also be the environmental sounds the animals produce themselves in a cage. Taken together, the results presented in the paper seem also consistent with the idea that the changes are driven by the rather random auditory background during the DE.

Although the study is based on previous work by the authors the main text should contain more information about the methods used. Currently, the reader would have to check the earlier publications in detail. Adding more information would thus make the study more accessible to the targeted broad readership.

E.g. (among others):

- it is not specified why correlations in a 100 μm range or that only 100 μm were analyzed until one sentence in the discussion.

- Is the response amplitude increase measured at the BF or at all significant frequencies? How was the bandwidth calculated? Could a decrease in bandwidth just be an ‘artefact’ of increased responses?

- How old were the mice? Where they properly aged matched? From the earlier work I would assume that they were. However, this is of particular importance as mice on a C57BL/6J background - as used in the current study - have shown to display frequency specific as well as unspecific age-related hearing loss.

Minor points:

Line 139 - 140: we assume that the surgery was done not completely in the dark (under infrared illumination) but rather with a surgical microscope in bright visible light? I suggest rephrasing that light was indeed used but should not have altered the conclusions.

Line 163 - 164: The given threshold is adequate for C57Bl6 mice. We suggest adding the background of the strain and e.g. the following reference: Zheng et al. Hear Res 1999 DOI: 10.1016/s0378-5955(99)00003-9.

Line 197 - 198: We assume that the word variance should not mean variance in the statistical sense but rather ‘variability’. This is confusing as the next sentence specifies calculation of standard deviation whereas the caption for Fig. 1F again uses ‘baseline variance’. Please revise.

Line 259 - 261: How was evoked response quantified exactly? Couldn't the lack of responsiveness be due to an increase in spontaneous activity, in other words a floor effect? Does the spontaneous activity correlate with the response amplitude? To make the assessment of spontaneous activity more accessible one could give the variability in ΔF/F.

Line 262 - 263: We suggest specifying that only peak evoked rates increased.

Line 277 - 278: According to the KS-Test presented in figure 2 response amplitudes increased for both L4 and L2/3 and did not ‘decrease slightly in L2/3’ as written? Please revise.

Line 280 - 283: As the authors have shown in previous work the threshold for sounds is decreased after DE. In the current study all animals were tested with the same sound level. Thus for DE animals this means testing higher above thresholds where tuning curves are wider. In consequence the bandwidth comparison is actually an underestimation of the true bandwidth decrease and might show decreased bandwidths in a larger frequency range as observed. This could be added to the discussion.

Are the cells actually responding to less tested frequencies? Is the change of bandwidth due to an increase at BF vs. decreased response at nonBFs or are both things happening? Is there a correlation between response amplitude and bandwidth? This could point towards possible mechanisms of the observed changes.

Line 401 - 402: The information why only a 100 μm range was studied should be contained in the manuscript. The field of view is 300 x 300 μm allowing for a maximum distance of roughly 400 μm. This is also not explained in the methods section. Longer distances could well be compared with the laser-scanning photostimulation. Therefore the very low likelihood of finding a correlation for on-responses beyond 100 μm should be cited (Winkowski & Kanold 2013).

Line 417 - 419: Wouldn't it be conceivable that for a given cell only a refinement i.e. changes of input strength for different frequencies from MGB to L4 without changing the set of MGB inputs to L4 occurs? We suggest rephrasing this sentence accordingly.

Line 439 - 441: How was it ensured that A1 was uniformly sampled? According to the table 1 only 2 - 3 fields could have been studied per animal. As A1 in mice is on the order of 800 x 1000 μm large about 9 imaging fields could have been chosen in A1 alone. Thus it is difficult for the reader to assess from the presented data that there was no sampling bias. Was there an initial gross scale mapping of A1 tonotopic gradient to indeed sample uniformly? How uniform between animals is the correlation of tonotopy and skull, MRI and CT based landmarks?

Line 447 - 449: The previous study has only compared ambient sound levels and not ambient sound spectra, right? If correct this should be acknowledged.

Fig. 1B: The scale bar is not specified in the caption. We assume it is 100 μm?

Fig. 1F: Is the magnitude of spontaneous activity given as variance - as suggested in the caption - or standard deviation? It would further be beneficial to give the data in units of ΔF/F.

Fig. 2A: Label 100 % (ΔF/F?). We suggest to schematically indicate the bandwidth measurement and response amplitude.

Fig 2B/D: The difference in response amplitude (B) as identified by the KS test does not seem to be driven by the frequency range identified in (D) 8 - 16 kHz as the mean ΔF/F seems rather low. The finding in D seems further driven by an unusually small mean response amplitude for 8 - 16 kHz in NR animals. Please comment.

Fig 3: Could the change of BF distribution be linked to biased sampling? How was the imaging area randomized?

Line 600 - 601: The citation should be updated.

Line 641 - 643: The citation should be updated. It has now been assigned a Volume, Issue and pages in Cerebral Cortex.

Line 653 - 654: The citation should be updated.

Line 662 - 663: The citation should be updated.
